# Nurse-led digital triage and health literacy navigation: redefining the front door of public health in the artificial intelligence era

**DOI:** 10.3389/fpubh.2026.1971194

**Published:** 2026-09-30

**Authors:** Wen Luo, Gui-hua Lu

**Affiliations:** Department of Hematology, Changhai Hospital, The First Affiliated Hospital of Naval Medical University, Shanghai, China

**Keywords:** digital triage, artificial intelligence, health literacy, nurse navigator, public health, human–AI collaboration

## Abstract

Artificial intelligence (AI)-driven digital triage is fast becoming the front door of public health systems worldwide. As algorithms take over symptom assessment and risk stratification, however, nurses risk being reduced to validators of machine output, and patients with limited health literacy risk being left behind. This perspective argues that nurses should not merely adapt to AI triage but redefine their role as “health literacy navigators,” positioned between algorithmic risk stratification and the patient who must act on it. We outline four functions that define the role: interpretation (translating AI output into language patients can use), empathy (understood as emotional calibration rather than reassurance), calibration (using clinical judgment to verify, challenge, or override algorithmic recommendations), and referral (guiding patients through the complex pathways that follow triage). The aim is a form of coevolution in which AI handles data-intensive stratification and nurses take on meaning-intensive navigation. Realizing this vision requires changes in nursing education, institutional policy, and AI system design. Embedding health literacy navigation in the digital triage workflow lets public health systems capture the efficiency AI offers without losing the relational core of nursing, strengthening resilience by ensuring that technological efficiency does not come at the expense of relational safety and health equity.

## 1 Introduction

The first contact many patients have with a health system is now with an algorithm. The United Kingdom’s NHS 111 online service routes users through artificial intelligence (AI)-powered symptom assessment (1), and evaluations of digitally supported telephone triage in England document both its promise and its safety challenges (2). Canada’s Info-Santé 811 helpline has integrated virtual pharmacy counseling into its nurse triage workflow (3), and systems elsewhere are deploying chatbots and risk-scoring tools to manage demand and direct patients to the appropriate level of care. The same transformation is unfolding across clinical specialties (4). An exchange that once began with a nurse listening, asking follow-up questions, and exercising clinical judgment now often begins with a dropdown menu. Digital triage has become the front door of public health.

The shift offers genuine advantages. AI triage is available at any hour, applies criteria consistently, and can shorten waits for patients who would otherwise delay care. But the focus on efficiency obscures a less visible change in what nurses are asked to do. Nurses were once the gatekeepers of triage, using clinical judgment, communication, and empathy to connect a patient’s distress to the system’s response. As AI takes over symptom evaluation and risk stratification, they increasingly confirm a decision the algorithm has already made, carrying out a process whose logic they did not shape.

This perspective argues that the rise of AI triage gives nursing an occasion to reassert, rather than retreat from, its core value. We use that term in a specific sense: the relational and interpretive work of attending to a particular person, making meaning of illness in that person’s circumstances, and carrying that understanding into safe and equitable action. Such work cannot be reduced to a risk score. Instead of accepting a diminished role downstream of the algorithm, nurses can position themselves as “health literacy navigators,” the human link between a machine-generated assessment and a patient who must understand it and act on it. When the first point of contact is algorithmic, the quality and equity of that encounter depend less on the algorithm’s sophistication than on the human infrastructure around it—and that infrastructure is nursing.

## 2 The problem: four dilemmas of AI-driven triage

Before proposing an alternative, it is worth examining where AI triage already falls short. Four problems stand out.

First, the accuracy of AI triage remains far from settled. A 2025 systematic review found that self-triage accuracy across symptom assessment applications ranged from 11.5 to 90.0%, whereas large language models ranged from 57.8 to 76.0% (5). A meta-analysis of ChatGPT reported pooled accuracy of 0.63 for GPT-3.5 and 0.86 for GPT-4, with substantial heterogeneity (6). These tools both overtriage and undertriage (7, 8), and head-to-head comparisons show physicians and nurses outperforming them on overall accuracy (9); even GPT-4 has well-documented limits on complex case challenges (10). AI-assisted Emergency Severity Index (ESI) systems may improve consistency (11), but they are trained on retrospective data and already meet considerable skepticism from the nurses expected to use them.

Second, digital triage alters the character of nursing work. In interviews with Swedish primary care nurses, text-based triage forced a wholesale revision of what the researchers called the “professional toolbox.” Nurses lost the “clinical ear”—the ability to read urgency in a caller’s tone, breathing, or hesitation (12). A separate chat-based pilot found nurses unsure how to weigh symptoms that appeared on intake forms but bore no relation to the patient’s stated complaint (13). Synchronous dialogue gave way to asynchronous writing, and the sensory cues that inform clinical judgment were reduced to typed text; nurses described being “one step further removed from the patient” (12). Trust in AI-based triage remains fragile on both sides of the screen (14), and patient safety in digital primary care still depends on nurses functioning as “fine-tuned instruments” who compensate for what the algorithm misses (15).

Third, digital triage can widen the health literacy gap rather than close it. The mechanism is cognitive load. Over the phone or in person, the nurse carries much of that load, asking follow-up questions, paraphrasing, and adjusting language in real time. A text-based or algorithmic tool hands the burden back to patients, who must read the prompts, understand the vocabulary, infer what a risk score implies, and decide what to do next; any one of those steps can fail. Health literacy—the knowledge and competence needed to access, understand, appraise, and apply health information—is exactly what these tools presuppose, and a systematic review finds that AI-driven health applications routinely fail users who lack it (16). When triage moves online without a human intermediary, digital exclusion becomes a gatekeeping mechanism that marginalizes the patients with the greatest need. The health literacy navigator fills this gap by taking back the cognitive load, converting algorithmic output into something a patient can act on, and restoring the exchange that makes clinical communication safe.

Fourth, the marketing of AI triage is quietly stripping nursing of its professional autonomy. When AI is sold as the solution to triage bottlenecks, nursing judgment is recast as a backup—a safety net rather than the core competency it is. The stakes concern patient safety, not merely professional pride. An algorithm cannot register a patient’s fear, read a family’s social context, or follow the thread of a health narrative that does not fit the template. When nurses experience AI triage as a threat to their autonomy, trust in the system erodes and safety suffers (14); when nursing judgment is devalued, patients lose the interpretive layer that makes triage meaningful.

## 3 The core proposition: nurses as health literacy navigators

Given these dilemmas, we propose a reframing: once AI has produced an initial risk stratification, the nurse should enter the workflow as a health literacy navigator, occupying the space between the algorithm’s assessment and the patient’s next move.

AI triage answers the “what” question—what might be wrong, and which risk category applies. Nurses are positioned to answer the “so what” and “now what”: what the score means for this person, what they should do next, and why. These are the questions that determine whether triage actually leads to care.

The role has antecedents in existing practice. Patient navigators have a documented history of guiding people through the care continuum and improving timeliness and screening completion (17, 18). Nurse navigators, in particular, have always built health literacy, spending time with patients, learning how they manage health, and identifying what they understand and where they struggle (19). Navigation programs have also proven cost-effective in conditions such as stroke (20). What has changed is the setting: in an era of algorithmic triage, navigation moves from a supplementary service to a necessary step in intake itself.

Our central claim is that the health literacy navigator is the product of coevolution between nursing and AI, not a defensive reaction to it. AI excels at data-intensive work; nurses excel at meaning-intensive work—figuring out what a score means for a specific person, motivating action, earning trust, and tailoring advice. The appropriate model is specialization rather than substitution, with the nurse as integrator who turns algorithmic output into real-world benefit. Studies of human–AI teams support this: teams outperform either party alone (21), and systems that know when to defer to a clinician improve diagnostic accuracy (22). The relevant design question is not how nurses adapt to AI but how AI should be built to support the work only nurses can do (Figure 1).

**Figure 1:**
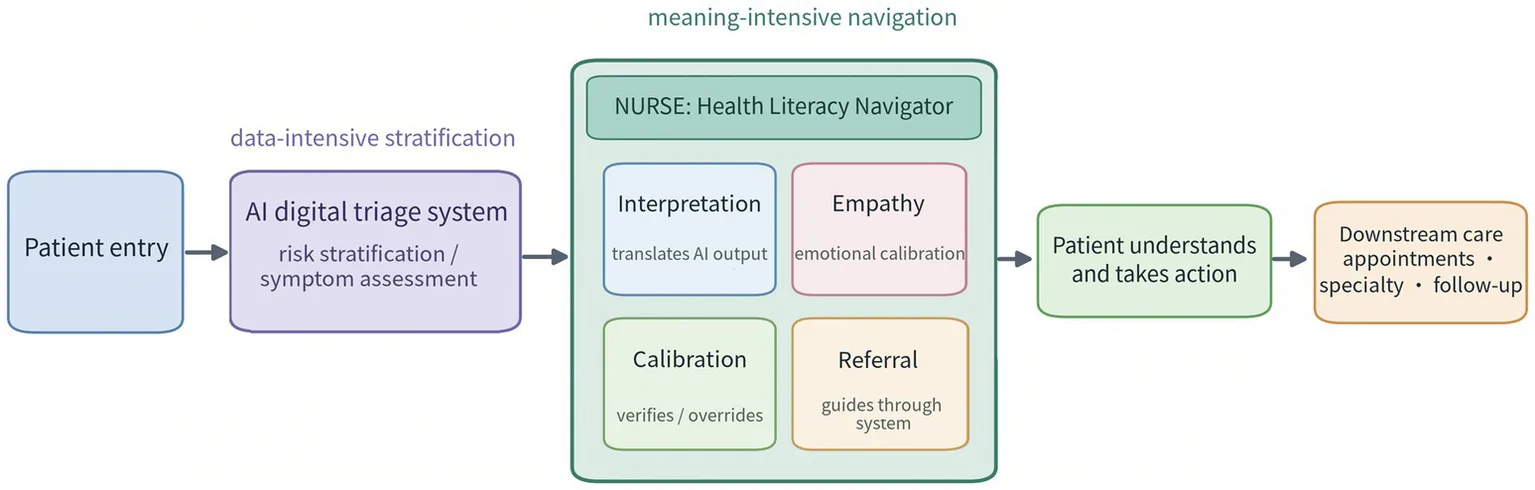
The nurse as health literacy navigator within the AI triage workflow. AI performs data-intensive risk stratification; the nurse performs meaning-intensive navigation through four functions, after which the patient reaches downstream services.

## 4 Four core functions of the health literacy navigator

The four functions that follow are not invented *de novo*. They synthesize three established lines of work: the patient- and nurse-navigation literature on guiding people through the care continuum (17–20); the health literacy tradition of accessing, understanding, appraising, and applying information (16); and human–AI complementarity research showing that teams outperform either party alone when systems defer appropriately to clinicians (21, 22). We mapped each function onto a specific limitation of AI triage identified in Section 2, so that every element of the role answers a documented gap rather than an abstract ideal. Table 1 summarizes this mapping before the functions are discussed in turn.

**Table 1:** The four core functions of the health literacy navigator and the AI triage limitations they address.

Navigational function	AI triage limitation addressed	Specific nurse actions	Value to patient/system
Interpretation	Opaque AI output that patients find hard to understand	Converts risk scores and recommendations into actionable language	Improves understanding and adherence
Empathy (emotional calibration)	AI cannot detect or regulate the patient’s emotional response	Tailors communication to emotional state: calming excessive anxiety and correcting false reassurance	Prevents emotion from blocking or delaying action
Calibration	Algorithmic bias and lack of clinical context	Verifies, questions, or overrides AI advice using clinical judgment	Safeguards clinical safety and health equity
Referral	AI can recommend but cannot guide patients through system complexity	Helps with appointments, transportation, insurance, and follow-up; links social resources	Turns triage advice into services actually received

### 4.1 Interpretation: translating algorithmic output into patient meaning

AI triage systems return risk scores, recommendation categories, and structured advice that remain opaque to most patients. A “moderate risk” label means little without clinical training, and “seek urgent care within 24 hours” often creates uncertainty rather than resolving it. The nurse’s task is to turn that output into something a patient can act on: why the algorithm recommends a given course, what the category actually implies in this situation, and what could happen if the patient does nothing. This matters most for patients with limited health literacy, for whom a misread instruction can mean the difference between timely care and a preventable emergency. Generative AI can produce readable plain-language materials, yet it also introduces inaccuracies, oversimplifies, and carries embedded biases (23); readability, in any case, is not the same as comprehension (16). The nurse checks both problems, verifying what the algorithm says with clinical judgment and supplying what it cannot say through empathic communication. Interpretation, in short, is the work of turning a score into a decision.

### 4.2 Empathy: bridging algorithm and humanity

AI processes data without registering the emotional consequences of its own output. It can flag a symptom as high risk while failing to recognize that the label may paralyze a patient with anxiety, or stamp “low risk” on symptoms the patient then dismisses. Empathy in this setting is better understood as emotional calibration: the ability to read how a patient is responding to an algorithmic result and bring that response to a point where action becomes possible. A terrified patient cannot absorb instructions; a falsely reassured one will not follow up. The navigator handles both, grounding the anxious patient in concrete next steps and pressing the dismissive one to take the result seriously. This cannot be scripted. It depends on reading a particular person in a particular moment, adjusting tone and pace, and knowing when to push and when to hold back. That capacity is a distinctly human asset and deserves protection as AI takes over the front end of triage.

### 4.3 Calibration: verifying and supplementing AI output with clinical judgment

AI triage systems have built-in limits: they train on retrospective data, lack the patient’s full medical and emotional context, and can systematically underserve underrepresented groups. The clinical stakes are high. Pulse oximetry algorithms embedded in automated vital-sign triage overestimate oxygen saturation in patients with darker skin pigmentation and can miss occult hypoxemia (24). An analysis of 3.5 million emergency visits found that Black patients received less follow-up than White patients with identical readings, a case of device bias compounded by clinician behavior (25). Algorithms trained largely on male-typical cardiac presentations can miss the atypical angina—fatigue, nausea, jaw pain—that women with acute coronary syndromes often report (26–28). The nurse’s calibration function is the counterweight, ensuring that coevolution proceeds on clinically sound terms rather than as resistance to automation. AI-assisted ESI triage shows promise (11), but it belongs in a decision-support role, not as a replacement for clinical judgment. Calibration calls for genuine AI literacy: recognizing an algorithm’s limits, challenging recommendations that do not fit the clinical picture, and overriding the machine when safety requires it. Consensus guidance calls for proactive evaluation of algorithmic bias across groups (29), and equity must be designed in from the start (30, 31). Nurses who routinely rubber-stamp algorithmic output are not performing the navigator function.

### 4.4 Referral: navigating system complexity after triage

Even accurate triage leaves patients facing a labyrinth of appointments, specialist referrals, diagnostic tests, follow-up visits, and insurance authorizations. The referral function guides patients through it—sorting out transportation, flagging missed appointments, connecting patients with financial help, and reassuring families when the system feels overwhelming. Oncology research shows that navigation addressing sociolegal barriers meaningfully improves treatment completion (32). In an AI triage workflow, referral marks the beginning of navigation rather than the end of the encounter. An algorithm can tell a patient to see a specialist within a week; it cannot find one who accepts their insurance, arrange a ride, or explain what to expect. These are the tasks that determine whether a triage recommendation ever becomes care.

We acknowledge a legitimate boundary question here. System navigation is sometimes treated as a separate, nonclinical occupation, and a dedicated health system navigator may be the right solution in large organizations. Our claim is narrower. At the triage front door, referral needs a clinical anchor—someone who understands why the recommendation was made and already holds the patient’s trust. The nurse is well placed to coordinate that work, partnering with social workers, scheduling staff, and community resources rather than personally absorbing every logistical task—owning the coordination without owning the entire burden.

## 5 Implementation pathways: education, policy, and practice

If health literacy navigation is to move from an idea to an operational role, three things must change together.

First, nursing education must rebuild its competency framework. Most curricula still assume in-person, analog care. Preparing nurses for AI-integrated triage means building competence in three domains: AI literacy (how triage algorithms work, where they fail, and what ethical risks, including bias, they carry); digital health communication (writing and speaking effectively in asynchronous, text-mediated settings); and health literacy assessment (quickly gaging what a patient understands and adjusting accordingly). A 2025 systematic review identified curriculum integration, faculty readiness, and ethics as the dominant themes (33), and others warn that nursing education must confront both the promise and the pitfalls of AI before it reaches the clinic (34). Chatbot tools are already entering nursing pedagogy (35, 36), yet surveys show faculty digital skills remain weak (37). WHO’s State of the World’s Nursing 2025 calls for nurses to be prepared to use digital technologies competently (38); health literacy navigation gives that call a specific, role-based form.

Second, institutions need policies that define the nurse’s authority in AI-integrated triage explicitly. These policies should guarantee that nurses can override an algorithmic recommendation when clinical judgment conflicts with it, that AI triage accuracy undergoes regular nurse-led audit, and that AI-assisted triage outcomes are tracked as nursing quality metrics. Consensus guidance stresses that clinical oversight and transparency must accompany algorithmic deployment (29). Without explicit protection, a nurse who challenges an AI recommendation risks looking obstructionist rather than responsible.

Third, the navigator model must be built and tested in real settings. The Swedish text-based triage experience is instructive: nurses assembled new professional toolboxes, but the process was uneven and stressful (12). Future systems should follow human-centered AI principles that support nurse autonomy rather than impose rigid scripts, and they should be codesigned with the nurses who will use them (39). That means including frontline nurses at the design table, building interfaces that augment rather than constrain clinical reasoning, and creating feedback loops for flagging errors. Pilots should be judged not only on wait times and throughput but also on whether patients understand the advice, whether they are satisfied, and, above all, whether equity holds.

A fair objection is whether this redefinition is feasible at all, or whether it merely adds work to an already stretched workforce. It need not do either if navigation is treated as substitution rather than addition. AI already absorbs transactional tasks—routine intake, standardized instructions, first-draft documentation—that once consumed nursing time, and navigation reorganizes what remains around the relational and interpretive work nurses already perform informally. It fails only where layered onto unchanged workloads without staffing, tools, or workflow redesign—which is why education, policy, and codesign are inseparable from it. Feasibility should be tested empirically: pilots can track nursing time and perceived burden alongside throughput and ask whether the time navigation consumes is offset by the work AI removes.

## 6 Conclusion

AI-driven digital triage is remaking the front door of public health, and the shift is unlikely to reverse. Nursing faces a genuine choice: accept a reduced role confirming algorithmic output, or redefine what the profession contributes to the new system.

We have argued for the latter. Positioning nurses as health literacy navigators—responsible for interpretation, empathy, calibration, and referral—reasserts what nursing does that algorithms cannot, at the moment when technology threatens to obscure it. These four functions are how algorithmic efficiency is converted into clinical effectiveness and health equity.

Realizing the vision requires coordinated work: curricula that prepare nurses for AI-integrated practice, policies that protect their authority to override the machine, and systems codesigned with the nurses who use them. Done well, AI does not diminish nursing; it frees nurses from transactional work and returns them to the profession’s defining purpose—attending to a particular person, making meaning of illness, and guiding that person through a fragmented system. The front door is being rebuilt, and nursing should help design it rather than simply stand behind it.

## Data Availability

The original contributions presented in the study are included in the article/supplementary material, further inquiries can be directed to the corresponding author.
